# A partially-open inward-facing intermediate conformation of LeuT is associated with Na^+^ release and substrate transport

**DOI:** 10.1038/s41467-017-02202-y

**Published:** 2018-01-15

**Authors:** Daniel S. Terry, Rachel A. Kolster, Matthias Quick, Michael V. LeVine, George Khelashvili, Zhou Zhou, Harel Weinstein, Jonathan A. Javitch, Scott C. Blanchard

**Affiliations:** 1 Department of Physiology and Biophysics, Weill Cornell Medicine, 1300 York Avenue, New York, NY 10065 USA; 2 Department of Psychiatry, Division of Molecular Therapeutics, Columbia University College of Physicians and Surgeons and New York State Psychiatric Institute, 1051 Riverside Drive, New York, NY 10032 USA; 3 HRH Prince Alwaleed Bin Talal Bin Abdulaziz Alsaud Institute for Computational Biomedicine, Weill Cornell Medicine, 1305 York Avenue, New York, NY 10021 USA; 4 Department of Pharmacology, Columbia University College of Physicians and Surgeons, 1051 Riverside Drive, New York, NY 10032 USA

## Abstract

Neurotransmitter:sodium symporters (NSS), targets of antidepressants and psychostimulants, clear neurotransmitters from the synaptic cleft through sodium (Na^+^)-coupled transport. Substrate and Na^+^ are thought to be transported from the extracellular to intracellular space through an alternating access mechanism by coordinated conformational rearrangements in the symporter that alternately expose the binding sites to each side of the membrane. However, the mechanism by which the binding of ligands coordinates conformational changes occurring on opposite sides of the membrane is not well understood. Here, we report the use of single-molecule fluorescence resonance energy transfer (smFRET) techniques to image transitions between distinct conformational states on both the extracellular and intracellular sides of the prokaryotic NSS LeuT, including partially open intermediates associated with transport activity. The nature and functional context of these hitherto unidentified intermediate states shed new light on the allosteric mechanism that couples substrate and Na^+^ symport by the NSS family through conformational dynamics.

## Abstract (web summary)

Neurotransmitter:sodium symporters (NSS) modulate the duration and magnitude of signaling via the sodium-coupled reuptake of neurotransmitters. Here the authors describe quantitative single molecule imaging of ligand-induced, functional dynamics of both intracellular and extracellular surfaces of LeuT, further defining the mechanism for NSS transport.

## Introduction

Neurotransmitter:sodium symporters (NSS) are integral membrane transport proteins that regulate the spatiotemporal parameters of neurotransmission through the reuptake of neurotransmitters from the synapse into the pre-synaptic neuron. Most eukaryotic NSS are comprised of 12 transmembrane segments (TMs), whereas prokaryotic homologs can have 12 or 11 TMs, with two inverted structurally related repeats (TMs 1–5 and TMs 6–10)^1,2^. These secondary active transporters couple the movement of substrates and Na^+^ across the bilayer, using the physiological Na^+^ gradient to concentrate substrates within the cell^3^. Their central role in neurotransmission is highlighted by the impact of small-molecule NSS inhibitors on mood and behavior. Clinically important antidepressants and psychostimulant drugs inhibit uptake by serotonin, norepinephrine, and dopamine transporters (SERT, NET, DAT), whereas amphetamines induce non-exocytic, transporter-mediated neurotransmitter release^4–6^. Crystal structures of prokaryotic homologs of human NSS^1,7^, and more recent structures of eukaryotic NSS^8,9^, have provided critical insights into the architecture of this essential protein family^10,11^, but the dynamics of the molecular events underpinning the transport mechanism remain obscure.

Substrate transport is thought to rely upon an “alternating access” mechanism^12^ in which conformational changes in the NSS alternately expose the Na^+^ and substrate-binding sites to opposite sides of the cell membrane^13,14^. Consistent with this mechanism, crystal structures of the 12 TM hydrophobic amino acid transporter LeuT from *Aquifex aeolicus* have been solved in both outward-open and inward-open conformations^7^, designations based on the extents of solvent accessibility to substrate and Na^+^-binding sites from the extracellular and intracellular surfaces, respectively. Structural studies have also revealed a substrate-bound, partially outward-open, but inward-closed, LeuT conformation, which is thought to represent an early stage in the transport cycle subsequent to substrate binding^1^. In contrast, functionally relevant intermediate conformations on the pathway to intracellular substrate release, in which LeuT’s intracellular side is partially open while the extracellular side is closed, have yet to be captured crystallographically.

To shed light on the order, timing, and regulation of molecular events occurring during NSS transport, we have established single-molecule fluorescence resonance energy transfer (smFRET) imaging techniques to quantify transport-related conformational changes in LeuT^15,16^. We employed detergent-solubilized protein for these studies to establish a close correspondence with the rich literature on structure and function of LeuT and other NSS transporters. We and others have reported that LeuT retains comparable binding activity^17–19^ and exhibits similar conformational states^20^ in detergent and liposomes. With this smFRET approach, we are able to perform time-dependent measurements of energy transfer efficiency between donor and acceptor fluorophores that are site-specifically attached to LeuT, and thereby monitor the changes in intramolecular distance between them, with sub-nanometer accuracy^21,22^. Utilizing these measurements, we have sought to elucidate transient, non-accumulating states that are difficult to capture using X-ray crystallography, as well as the sequence and timing of conformational transitions that are potentially masked by ensemble averaging. In addition, we have also sought to quantify the allosteric coupling between the conformational changes observed by smFRET and the binding of various ligands in terms of thermodynamic coupling, which manifests as either changes in the occupancy of states or the rate of transitions between those states^23^.

In previous smFRET investigations of LeuT, we have shown that movement of its intracellular N-terminal segment away from the 12 TM bundle is associated with substrate-modulated intracellular gating^15,16^. Rearrangements of this nature have been corroborated by electron paramagnetic resonance (EPR) spectroscopy, which evidence a comparable movement of the N terminus and an associated movement of the contiguous TM1a of smaller amplitude^24^. X-ray crystallography studies of a mutant LeuT construct with a disrupted intracellular gate show a much larger displacement of TM1a^7^, which was recapitulated by EPR^25^. Our smFRET measurements further revealed that substrate-induced intracellular gating in LeuT is associated with closure at its extracellular surface^15^, a result supported by recent hydrogen-deuterium exchange experiments^20^. However, limitations stemming from low signal-to-noise ratios (SNR) and rapid fluorophore photobleaching prevented the unambiguous identification of distinct FRET states at the extracellular surface and thus hindered determination of the impact of Na^+^ and amino acid substrates on extracellular gating dynamics, as well as an evaluation of the extent to which extracellular and intracellular gating are coupled.

Recent technical advances in dye photochemistry have greatly improved fluorophore performance^26,27^ and the implementation of scientific CMOS (sCMOS) camera technologies have substantially improved the SNR of smFRET imaging and dramatically increased the number of molecules that can be imaged simultaneously^22^. Together, these advances now enable us to identify and quantify transitions between distinct extracellular conformations in LeuT for the first time. We show here that the observed coupling between the intracellular and extracellular sides of LeuT is weaker than expected from a model in which the two sides undergo rigid motion and exhibit strictly alternating solvent accessibility. Importantly, we observe partially open intermediate conformations of both the extracellular and intracellular regions, which represent transitional states in the alternate access cycle. The abundance of the partially open inward-facing intermediate state of LeuT, which has not yet been captured by X-ray crystallography, is specifically increased by the transported substrates alanine and Na^+^, and can be depleted in the presence of high concentrations of Na^+^. We show that this partially open inward-facing intermediate represents a conformation associated with Na^+^ release from the Na2 site that is directly on path to intracellular substrate release.

## Results

### LeuT samples three intracellular and extracellular conformations

**Fig. 1:**
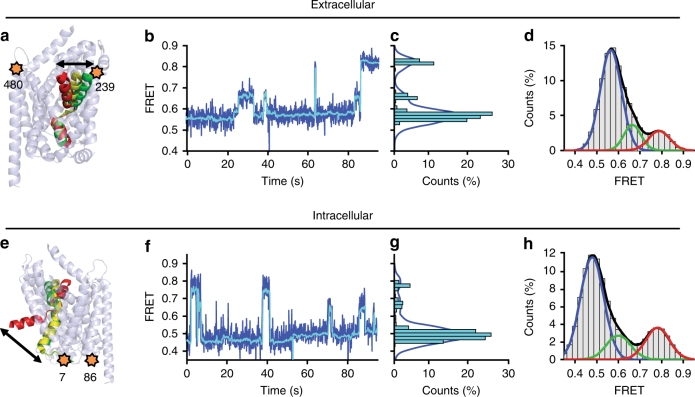
Detection of intermediate-FRET states at extracellular and intracellular sides of LeuT. **a** Cartoon representation of LeuT showing the extracellular-labeling sites K239C and H480C as orange stars with TM11 and EL3 hidden for clarity. The arrow highlights the axis of motion expected in FRET experiments from relative positions of TM6a in crystal structures with closed (red, 3TT3), intermediate (yellow, 2A65), and open (green, 3TT1) extracellular sides. **b** Representative raw (blue) and filtered^50^ (cyan) smFRET trace from the extracellular side (K239C/H480C) in the absence of Na^+^ and substrate. **c** Histogram of the FRET values in **b**. **d** Histogram of all FRET values in all traces (gray bars), fitted with a Gaussian distribution for each FRET state (colored lines), along with the summed model distribution (black line). **e**–**h** As in **a**–**d**, but monitoring FRET from the intracellular perspective (H7C/R86C) and the movements of the N terminus

To enable high spatial and temporal resolution imaging of conformational changes at the extracellular side of LeuT, the double-cysteine mutant K239C/H480C (located at the extracellular ends of TM6a and TM12, respectively) was site-specifically labeled with the intra-molecularly photostabilized LD550 and LD650 FRET pair^26,28^ (Fig. 1a; Supplementary Fig. 1a). With the protein thus labeled and using sCMOS cameras^22^, we observed a nearly twofold increase in SNR relative to previous experiments^16^ (Supplementary Fig. 1b), and wild-type ligand-binding affinities and Na^+^-response characteristics (Supplementary Fig. 1b–g). The enhancements also enabled simultaneous increases in temporal resolution (100 ms vs. 160 ms) and throughput (7500 vs. 200 raw FRET traces per movie).

Unbiased model discovery of the higher quality data using ebFRET^29^ revealed evidence of three distinct states on both sides of LeuT (Supplementary Figs. 2 and 3). Subsequent idealization of individual smFRET recordings using these initial parameters was performed using hidden Markov Modeling methods implemented in the SPARTAN software package^22^.

Wide-field smFRET imaging of LeuT with sCMOS cameras revealed three distinct extracellular conformations exhibiting low (~0.55), intermediate (~0.67), and high (~0.79) FRET values, which interconverted spontaneously in the absence of substrates (Fig. 1b–d; Supplementary Fig. 2a). Based on the direction and magnitude of TM6a motions inferred from distinct LeuT crystal structures^1,7^ (Supplementary Table 1), we considered the interconversion of these states to reflect changes in the position of TM6a relative to TM12 that correspond to relatively open, intermediate, and closed states, respectively (Fig. 1a). In accordance with these distinct degrees of extracellular gate opening, three FRET states were also observed in LeuT site-specifically labeled in a similar fashion at positions A309C and H480C, which report on distance changes between EL4 and TM12 (Supplementary Fig. 4; Supplementary Table 1).

In parallel, we examined the dynamic processes at the intracellular side of LeuT by site-specific labeling of the double-cysteine mutant H7C/R86C (located immediately N-terminal to the start of TM1a and in IL1, respectively; Fig. 1e). In line with inward-closed and inward-open LeuT conformations^1,7^, LeuT labeled at these positions exhibited distinct low-FRET and high-FRET states that represent open and closed positions of the N terminus relative to IL1, respectively^15,16^ (Fig. 1e). Here, the higher data quality afforded by the LD550/LD650 FRET pair and sCMOS cameras notably revealed evidence for three distinct FRET states: low-FRET (~0.47), intermediate-FRET (~0.63), and high-FRET (~0.79) (Fig. 1f–h; Supplementary Fig. 2b). In the absence of substrates, sampling of the newly identified intermediate-FRET state was relatively rare, representing only ~13% of the time-averaged population.

### Na^+^ compacts LeuT’s intracellular and extracellular surfaces

**Fig. 2:**
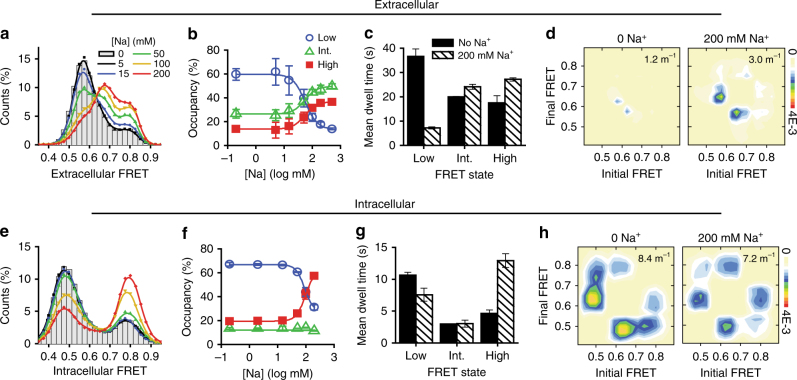
Na^+^ promotes higher FRET states at the extracellular and intracellular sides. **a** FRET values from all traces were summed into histograms for experiments imaging extracellular labeled LeuT in the absence of Na^+^ (bars) or in the presence of increasing concentrations (lines). **b** Fraction of dwell times in the low- (blue circle) intermediate- (green triangle), and high-FRET (filled red square) (extracellular states obtained from hidden Markov modeling. **c** Mean dwell times in each of the assigned states in the absence (solid bars) and presence of 200 mM Na^+^ (shaded bars). **d** Transition density plots, which display the mean FRET values in the dwell before (*x*-axis) and after (*y*-axis) each transition, show the specific number and type of transitions (per second rate scale at right), in the absence of Na^+^ (left) and in the presence of 200 mM Na^+^ (right). **e**–**h** As in **a**–**d**, but for the intracellular-FRET pair. Lines in **b** and **f** are fits to dose–response functions with Hill coefficients fixed at 2.0 and EC_50_ of 55 mM and 130 mM, respectively. Error bars in all panels are the mean ± s.d. of two independent experiments, including a total of 9255 and 11,169 molecules for extracellular and intracellular perspectives, respectively

To clarify the functional significance of the three FRET states observed at the extracellular and intracellular surfaces of LeuT, as well as the roles they play in the transport mechanism, we first examined the impact of Na^+^ binding. At the extracellular side (K239C/H480C), the addition of Na^+^ decreased low-FRET state occupancy in a concentration-dependent manner, favoring intermediate-FRET and high-FRET states with equal probability (Fig. 2a, b). The observed redistribution of FRET states principally resulted from a nearly four-fold destabilization of the low-FRET conformation (Fig. 2c), and a modest increase in overall dynamics (Fig. 2d). Similar effects were observed when we measured FRET between EL4 and TM12 (A309C/H480C; Supplementary Fig. 4). These findings suggest that Na^+^ depopulates the fully open extracellular configuration in which TM6a and EL4 are positioned furthest from TM12, to stabilize instead a narrowed extracellular vestibule.

Consistent with the compaction of the extracellular side, pre-incubating LeuT with Na^+^ dramatically slowed the rate at which the bulky leucine (Leu) substrate was able to bind (Supplementary Fig. 5a). By contrast, the smaller amino acid alanine (Ala), which is much more rapidly transported^17,30^, showed no such delay in binding (Supplementary Fig. 5b). Together with ^3^H-Leu association experiments with the S2 site mutant, LeuT-L400S, and the S1 site mutant, LeuT-F253A^16–18^ (Supplementary Fig. 5c, d), these data suggest that Ala retains ready access to both the S1 and S2 binding sites in the narrowed intermediate-FRET extracellular conformation, while access of the bulkier Leu substrate to the S1 site is selectively hindered. Hence, we infer that Leu binding to the deeper primary binding site requires an opening of the extracellular vestibule, which occurs less frequently in the presence of bound Na^+^ (Fig. 2a–d). These data argue that conformational changes in LeuT associated with Na^+^ binding may contribute to substrate selectivity in transport.

Consistent with our previous smFRET experiments^15,16^, and more recent reports^25,31,32^, the binding of Na^+^ also closed the intracellular side of LeuT, increasing the high-FRET state dwell time, but also destabilizing the inward-open, low-FRET configuration (Fig. 2e–h). Notably, Na^+^ binding had no detectable impact on the relatively rare intermediate-FRET intracellular configuration. Hence, Na^+^ binding both prepares LeuT for selective substrate binding at the extracellular surface and closes the intracellular gate to prevent premature Na^+^ transport and/or channel formation.

### Ala promotes intracellular gating via intermediate states

As described previously^15,16^, the effects of amino acid binding were analyzed in the presence of 5 mM NaCl, a concentration of the coupling cation that enables substrate binding but also facilitates transport-like inward release of substrate by mimicking the low cytoplasmic Na^+^ concentration in vivo^15,16^. This experimental setup allows for dissociation and minimal rebinding of Na2 and the subsequent allosteric triggering of inward opening and release of substrate^16,17^. Under these conditions, and with the benefit of increased experimental throughput and SNR, we examined the effects of the rapidly transported Ala substrate^30^ on the dynamics in LeuT.

**Fig. 3:**
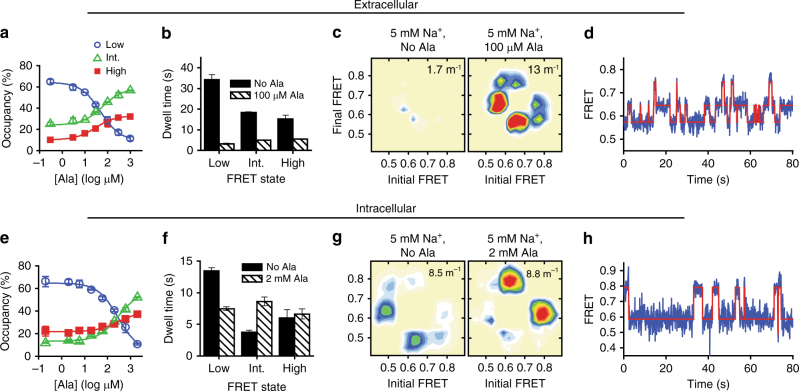
Ala alters dynamics at both sides and promotes an intermediate inward-open conformation. smFRET experiments were performed in the presence of 5 mM Na^+^. **a** Fractions of time in the low- (blue circle), intermediate- (green triangle), and high-FRET (filled red square) extracellular states. **b** Transition density plots, from experiments in the absence (left) and presence of 100 μM Ala (right). The average transition rate is shown in the upper-right corner of each plot. **c** Mean dwell times in each FRET state in the absence (solid bars) and presence (shaded bars) Ala. **d** Representative smFRET trace showing the behavior in the presence of Ala (compare to Fig. 1b). **e**–**h** As in **a**–**d**, but with the intracellular-FRET pair. Lines in **a** and **e** are fits to a dose–response function with Hill coefficients fixed at 1.0 and EC_50_ of 48 μM and 270 μM, respectively. Ala concentrations in **b**–**d** and **f**–**h** were chosen to compensate for this difference in apparent affinity. Error bars in all panels are the mean ± s.d. of two independent experiments, including a total of 15,002 and 10,769 molecules for extracellular and intracellular perspectives, respectively

Ala increased both intermediate-FRET and high-FRET state occupancy at the extracellular surface, while depopulating the low-FRET state (Fig. 3a) similar to the effect of saturating Na^+^ alone (Fig. 2b). The observed changes were, however, greater than for saturating Na^+^ alone, and resulted from an ~11-fold increase in the rate of exiting low-FRET (Fig. 3b, c). Critically, while Na^+^ alone did not alter the FRET lifetimes of the intermediate-FRET and high-FRET states (Fig. 2c), the addition of Ala led to four to eight-fold decreases in the lifetimes of these states (Fig. 3b). These changes to the energy landscape dramatically increased the overall rate of dynamics (Fig. 3c, d). Similar Ala-induced increases in dynamics were observed when FRET was measured between EL4 and TM12 (Supplementary Fig. 4c, d). These findings suggest that extracellular intermediate-FRET and high-FRET states of LeuT primarily reflect conformations that are allosterically coupled to Ala binding.

At the intracellular side of LeuT, Ala binding increased both intermediate-FRET and high-FRET state occupancy (Fig. 3e), in contrast to the impact of Na^+^ alone, where the principle effect was stabilization of the high-FRET state (Fig. 2e–g). Ala also specifically increased the frequency of transitions between intermediate-FRET and high-FRET states, which were relatively rare in the presence of Na^+^ alone (Fig. 3f–h). Such changes primarily arose from transition paths between intermediate-FRET and high-FRET being favored over low-FRET states (Fig. 3g; Supplementary Fig. 6). Here, the overall rate of intracellular dynamics now appear similar in the absence and presence of Ala, whereas we previously observed an overall increase in dynamics^16^. This distinction arises from our present capacity to detect spontaneous dynamics, which were previously hidden, between low-FRET and intermediate-FRET states in the absence of substrate. Similar to our results on the extracellular surface, these observations collectively suggest that the intracellular configuration exhibiting intermediate-FRET reflects a conformation of LeuT that is allosterically coupled to Ala binding.

To evaluate the functional significance of the observed substrate-dependent impacts on LeuT dynamics, we performed analogous steady-state measurements in the presence of the slowly transported substrate Leu^30^. The addition of Leu in the presence of 5 mM Na^+^ depopulated the low-FRET extracellular LeuT configuration (Supplementary Fig. 7a, b) by reducing the low-FRET state lifetime more than fivefold and slightly increasing the lifetimes of intermediate-FRET and high-FRET states (Supplementary Fig. 7c, d). These effects were globally similar to saturating Na^+^ alone (Fig. 2a–d), but in stark contrast to the rapid dynamics and shorter intermediate-FRET and high-FRET state lifetimes in the presence of Ala (Fig. 3b–d). Consistent with Leu’s higher binding affinity to LeuT^17,30^, these impacts exhibited a  > 150-fold lower EC_50_ compared to Ala (0.28 μM for Leu vs. 48 μM for Ala).

As observed previously^16^, Leu reduced spontaneous intracellular gating dynamics and depopulated the low-FRET state by specifically stabilizing the high-FRET, inward-closed LeuT configuration (Supplementary Fig. 7e–h). These impacts were similar to saturating Na^+^ alone (Fig. 2e–h), and in stark contrast to the increased transitions between intermediate-FRET and high-FRET states observed in the presence of Ala (Fig. 3e–h). Notably, and also in marked contrast to Ala, Leu had no effect on the intracellular intermediate-FRET state, which remained weakly populated (~13%). These results suggest that the allosteric coupling of the intracellular intermediate-FRET state to substrate binding is also substrate-specific, and reflects the differences between the measured transport efficiencies of the two ligands^30^.

### Partial intracellular opening is associated with transport

To further distinguish conformational changes associated with substrate binding that are independent of transport from those that are necessary for (or facilitate) transport, we performed analogous measurements in the presence of Li^+^ rather than Na^+^. In the presence of Li^+^, Ala can bind to LeuT but is not transported due to Li^+^’s distinct binding mode and smaller ionic radius^16^. Although the EC_50_ of Li^+^ for inducing high-FRET state stabilization was seven-fold higher than for Na^+^ (~400 mM vs. 55 mM), Li^+^ alone had broadly similar effects as Na^+^, favoring extracellular intermediate-FRET and high-FRET states (Supplementary Fig. 8a–d). Li^+^ also specifically promoted an intracellular high-FRET state, while having little to no effect on intermediate-FRET state occupancy (Supplementary Fig. 8e–h).

**Fig. 4:**
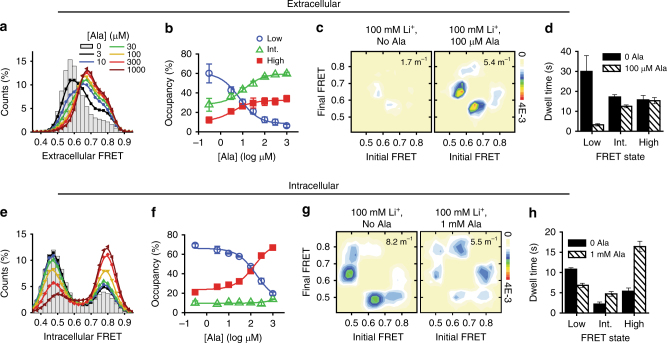
Li^+^ supports Ala-induced dynamics and formation of the intermediate-FRET state only at the extracellular side. **a** FRET histograms of extracellular labeled LeuT in the presence of 100 mM Li^+^ and the indicated concentration of Ala. **b** Fraction of time in the extracellular low- (blue circle), intermediate- (green triangle), and high-FRET (filled red square) states. **c** Transition density plots in the absence (left) and presence of 100 μM Ala (right). **d** Mean dwell times in each FRET state without (solid bars) and with (shaded bars) Ala. **e**–**h** As in **a**–**d**, but imaging with the intracellular-FRET pair. Lines in **b** and **f** are fits to dose–response functions with Hill coefficients fixed at 1.0 and EC_50_ of 7.7 and 170 μM, respectively. Error bars in all panels are the mean ± s.d. of two independent experiments, including a total of 15,287 and 11,978 molecules for extracellular and intracellular perspectives, respectively

Analogous to our substrate binding studies in the presence of Na^+^, we probed the impact of Ala on LeuT dynamics in the presence of 100 mM Li^+^, a concentration that is substantially below saturation, but sufficient to support observable substrate binding^16^. Consistent with Li^+^-supported substrate binding, and the effects observed in the presence of both Na^+^ and Ala (compare Figs. 3c and 4c), the rates of dynamics on the extracellular surface of LeuT increased threefold with Ala (Fig. 4a–d). However, in stark contrast to the dynamics observed in the presence of both Na^+^ and Ala (Fig. 3e–h), and in line with the inability of Li^+^ to support transport, the addition of Ala in the presence of Li^+^ stabilized the intracellular high-FRET state, while having little to no effect on the transition rates between intermediate-FRET and high-FRET states (Fig. 4e–h). Hence, we observed no enrichment of the intermediate-FRET configuration of the intracellular side of LeuT in the presence of Li^+^.

Given that Li^+^ supports binding of two Ala molecules per LeuT molecule but not transport^16^, we conclude that Li^+^ cannot support the substrate-induced, allosteric modulation of the intracellular gating essential for transport^23,33^. The reduction in dynamic transitions on the intracellular side of LeuT, and the lack of enrichment of the intermediate-FRET configuration, suggests that the intracellular intermediate-FRET state corresponds to an inward-facing configuration that must be traversed in order for transport to occur. As mutations that disrupt Ala binding to either the S1 or S2 sites prevent both Ala transport^17^ and Ala-induced intracellular gating dynamics^16^, we infer that specific configurations of the S1, S2, and Na^+^ sites must be established in order to promote the intermediate-FRET intracellular conformation associated with transport.

### Partial inward opening is coupled to Na2 release

We next sought to discern the structural features of the intracellular intermediate-FRET state populated by the presence of Na^+^ and Ala, and to identify which specific steps in the transport process require LeuT to take on this conformation. In particular, we hypothesized that sampling of the intracellular-FRET state may relate to intracellular structural rearrangements and solvation, and the release of Na^+^ from the Na2 site—events that are thought to precede substrate release^17,34–36^. Markov State Model (MSM) analysis of microsecond timescale, unbiased simulations of the homologous human dopamine transporter (hDAT) have revealed spontaneous transitions from inward-closed states to such states of intermediate inward opening[35]. These hDAT intermediate states are characterized by (i) displacement of the N terminus, (ii) outward motion of TM1a, (iii) unwinding of the intracellular region of TM5, and (iv) water penetration through the opened intracellular gate that exposes the Na2 site to intracellular water. Interestingly, these states appear with higher probability immediately before, during, and after Na^+^ is released from the Na2 site^35^. While LeuT is naturally expressed in a hyperthermophilic bacterium, and thus is too rigid at room temperature for unbiased MD simulations to spontaneously sample such intermediates, we hypothesized that the partially inward-open conformations observed in our simulations of hDAT are analogous to the intracellular intermediate-FRET state observed in our smFRET experiments of LeuT.

In order to compare directly these inward-facing MSM macrostates of hDAT to the states observed with smFRET, we used them as templates to construct an ensemble of corresponding structural models of LeuT (Supplementary Fig. 9 and Methods). The inward-facing templates were: Na2-bound (Model State 2); Na^+^ displaced from the Na2 site and bound in a more intracellular site (Model State 3); and Na2-released (Model State 4). In addition, we built an inward-occluded model of LeuT from the hDAT occluded state, bound to both Na^+^ and substrate (Model State 1). Notably, the occluded LeuT model reproduced the distance characteristics of the inward-closed structure of LeuT (Supplementary Fig. 9d), and the inward-facing intermediate LeuT models reproduced the unwinding of the intracellular end of TM5 (Supplementary Fig. 9f) observed in both hDAT^35^ and MhsT^34^. Distance distributions predicted computationally from these models show that in the inward-facing intermediate state models (Model States 2–4), the distance between H7 and R86 increases by ~4–6 Å compared to the inward-closed model (Model State 1) and the crystal structure of the occluded state of LeuT^1^ (Supplementary Fig. 9a–e). This displacement is consistent with the transition from the intracellular closed, high-FRET to inward-facing, intermediate-FRET (Supplementary Table 1).

Based on the structural similarity between intermediate-FRET state sampled by LeuT and the partially inward-facing state sampled by hDAT, we reasoned that the FRET state was likely to be involved in the same functional process as the state identified in our unbiased MD simulations: Na^+^ release from the Na2 site. We should then find experimentally that saturating Na^+^ would drive the Na^+^ release process in reverse, leading to reduced occupancy of the intermediate-FRET state (Model States 2–4) in favor of the high-FRET state (Model State 1). Thus, to further characterize our metastable intermediate state we performed smFRET experiments in which the Na^+^ concentration was varied in the presence of a constant concentration of Ala (250 µM).

**Fig. 5:**
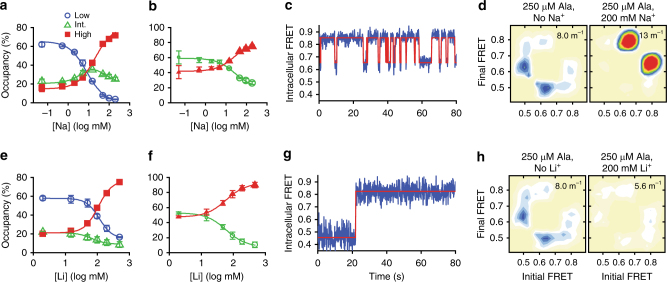
Effect of Na^+^ in the presence of Ala on intracellular dynamics. **a**–**d** smFRET imaging of intracellular-labeled (7 C/86 C) LeuT was performed in the presence of 250 μM Ala and the indicated concentration of NaCl. **a** Average occupancies in low- (blue circle), intermediate- (green triangle), and high-FRET (filled red square) states were fit to dose–response functions with EC_50_ values of 8–20 mM. **b** Relative occupancies of the intermediate-FRET and high-FRET states, with symbol sizes scaled to reflect the overall occupancy in these states. **c** Representative smFRET trace (blue) and idealization (red) in the presence of Ala and 200 mM Na^+^. **d** Transition density plots, with the concentration of Na^+^ shown above each plot. **e**–**h** Experiments were performed as in **a**–**d**, but with the indicated concentrations of LiCl instead of NaCl. **e** State occupancies were fit to dose–response functions with EC_50_ values of 110, 67, and 100 mM, respectively, and Hill coefficients fixed at 2.0. Error bars are the mean ± s.d. of two independent experiments, including a total of 25,119 (**a**–**d**) and 12,359 molecules (**e**–**h**)

As expected, increasing concentrations of Na^+^ led to decreased occupancy of the intracellular low-FRET state in favor of both intermediate-FRET and high-FRET states (Fig. 5a). Focusing on the intermediate-FRET and high-FRET states, which we hypothesize correspond to substrate-bound states enabled by the binding of Na^+^, the occupancy of the intermediate-FRET state is found to be first enhanced at lower Na^+^ concentrations and then depleted at the highest concentrations (Fig. 5a, b), consistent with the prediction that saturating Na^+^ should reverse the Na^+^ release process to favor occupancy of the occluded state. Notably, in the presence of Ala, the intermediate-FRET state is rapidly sampled even in the presence of saturating Na^+^ concentrations (Fig. 5c, d), suggesting that the transition to the intermediate-FRET state does not require Na2 release, but dwells in this state are brief (Supplementary Fig. 10). These findings suggest that the intermediate-FRET state is less stable while Na2 is bound, and prone to transition back to an inward-closed state, but that it is relatively stable after Na^+^ is released from the Na2 site. These results are consistent with a model in which the intermediate-FRET state is occupied during the process of Na2 release.

We note that in contrast, when Li^+^ substitutes for Na^+^, increasing ion concentrations in the presence of Ala (a condition that supports Ala binding but not its transport^16^) led to a clear decrease in both intracellular dynamics and intermediate-FRET state occupancy without any enhancement at lower Li^+^ concentrations (Fig. 5e–h). These results suggest that (i) occupancy of the intracellular intermediate-FRET state is enhanced by Ala and a single Na^+^ bound, likely in the Na1 site, where it remains bound in the partially inward-open states observed in our hDAT simulations, and (ii) conditions that do not support the stabilization of the intracellular intermediate-FRET state do not support efficient transport. Thus, we conclude that the intermediate-FRET state is a transport intermediate state from which Na^+^ is released from the Na2 site, on the pathway to substrate release, and allosterically stabilized by efficiently transported substrates.

## Discussion

The elucidation of substrate-dependent dynamic processes at the extracellular and intracellular sides of LeuT enables us to present a model of the NSS transport mechanism that builds on our previous findings^15–17^ to include heretofore unresolved, metastable intermediate states that serve clear functional roles. These intermediate states represent critical transitions between open and closed conformations at both the extracellular and intracellular sides of LeuT.

In the absence of Na^+^ and substrates, we observed slow, reversible conformational transitions between high-FRET, fully closed and low-FRET, fully open states at both sides of LeuT, on the minutes timescale. Notably, LeuT exhibited 60–70% low-FRET state occupancy on both surfaces in the absence of Na^+^ and substrate. This observation suggests that apo LeuT may adopt “open” conformations at both sides of the membrane simultaneously, at least some fraction of the time. Such states are not part of classical alternating access^12^ or more contemporary rocker-bundle mechanisms^2^. Hence, the smFRET data suggest that the extracellular and intracellular sides of LeuT are not constrained by an obligatory coupling. Indeed, we estimate from the experimental data (Methods section) that the maximum allosteric destabilization^37^ of the apo state in which LeuT is both outward-open and inward-open simultaneously is on the order of 0.4 kT. Thus, the increase in the energy cost of opening one gate when the other is already in an open conformation is less than the cost of breaking a single hydrogen bond, making such a conformation readily accessible under thermal fluctuations.

Allostery is thought to play a central role in the transport mechanism^23,33^, but it remains poorly understood how transitions between functionally relevant conformational states are thermodynamically coupled to processes comprising the transport cycle, such as substrate binding and release. We find that the allosteric modulation of state occupancy and transition rates by Na^+^ and substrates is an essential component of the LeuT transport cycle. The binding of Na^+^ allosterically stabilizes an inward-closed conformation of LeuT and also reduces both the lifetime, and the likelihood of entering, the fully open extracellular state. Compaction of the intracellular gate represents a transport-inhibited conformation^15,16^, which likely serves to prevent uncoupled Na^+^ translocation (Na^+^ uniport)^23^ that might otherwise have been facilitated by the states described above in which both sides of LeuT may be “open” simultaneously. Compaction of the extracellular vestibule by Na^+^ binding also slows access of the bulky, poorly transported Leu to the S1 site (Supplementary Fig. 5), while the more efficiently transported Ala can still bind readily. In vivo, where the external Na^+^ concentration is saturating, such kinetic discrimination in binding rates is expected to contribute to substrate selectivity in the transport mechanism. The binding of the transported substrate also contributes to inducing extracellular gate closure by further destabilizing outward-open LeuT configurations.

Classical alternating access models^12^ imply that the binding of ligands induces opposing structural changes at the extracellular and intracellular sides. The improvements in imaging we have achieved here have made it possible to examine quantitatively the extent to which substrate-induced conformational processes at the extracellular and intracellular sides of LeuT display such inverse coupling. At the extracellular side, binding of substrate and/or ions generally induces dynamic sampling of intermediate and closed states, even under conditions that do not support transport, such as the substitution of Li^+^ for Na^+^ or the presence of the poorly transported substrate Leu. By contrast, frequent transitions from the closed to intermediate intracellular configurations only occur under conditions that support transport, namely the presence of Na^+^ and Ala. Thus, substrate-induced and ion-induced occupancy of extracellular closed states appear necessary but not sufficient for transport, while transitions between closed and intermediate states at the intracellular side are likely on path to substrate release. The observed differential regulation of dynamics on the extracellular and intracellular sides further supports the conclusion that these regions do not display an obligatory coupling but rather have the capacity to move as relatively distinct microdomains that are orchestrated through the allosteric effects of occupation of the S1, S2, Na1, and Na2 sites^16,17,23^.

Homology modeling of LeuT based on previously reported partially inward-open conformations of hDAT that are associated with Na^+^ release from the Na2 site^35^ suggests that the intracellular intermediate-FRET state is consistent with such conformations. Furthermore, while Ala-induced enhancement of the intracellular intermediate-FRET state requires Na^+^, we find that saturating Na^+^ reduces the enhancement and shortens dwell times in the intermediate state. Both the computational modeling and this non-linear Na^+^ dependence suggests that Ala-induced, partial inward opening is allosterically coupled to the release of Na^+^ from the Na2 site. In support of this contribution of the Na2 site, mutations that disrupt this site interfere with Na-induced intracellular closure^31^. In addition, previous investigations have shown that inward release of substrate from the S1 site is dependent on the release of Na^+^ from the Na2 site^17^. These findings lead us to propose that the inward-facing intermediate state of LeuT, observed here to be induced by the efficient substrate Ala, corresponds to a functionally relevant, partially open, intermediate state associated with Na^+^ release from the Na2 site that necessarily precedes the release of the Na^+^ from Na1 site and the substrate from the S1 binding site.

Together, these findings provide specific, concrete evidence for metastable states with intermediate degrees of intracellular gate opening transited during functional steps in the transport mechanism. Our findings also assist in providing a functional context for interpreting a variety of other structural observations in the literature. For example, while it was not commented upon, a low occupancy state with an intermediate degree of inward opening can be discerned in EPR measurements utilizing the same labeling sites^25^. The inward-facing occluded conformation captured by the crystal structures of MhsT, in which the occupied Na2 site was partially solvated from the intracellular side^34^, may cautiously be considered to represent either some structural features of the intracellular intermediate-FRET state, or to directly precede it^36^. Finally, fluorescence quenching measurements^38^ have suggested that substrate-dependent intracellular rearrangements may occur after substrate binding but before inward release. As the quenching signal detected in that study was observed in the presence of saturating Na^+^ concentrations, and quenching was of lesser magnitude in the presence of the well transported Ala than the poorly transported substrate Leu, the reported rearrangements likely precede transitions to the intracellular intermediate-FRET state observed here, which are specifically promoted only by Na^+^ and Ala.

While a number of intermediate-like LeuT conformations have also been observed in biased molecular dynamics simulations^17,39–41^, these are difficult to interpret in a functional context as they have yet to be validated experimentally as metastable states, or shown to have properties that can be associated with a specific step in the transport cycle. Such associations, which we have endeavored to establish through the present investigations, are essential for the identification of true intermediate states.

As previously described^16,17^, transport-related events are expected to require the presence of a second amino acid substrate bound in the S2 site of LeuT. Future investigations will be needed to delineate both the role of S2 in the transition to the inward-intermediate state and subsequent release of Na1 and S1, as well as the roles of the observed gating dynamics in transporter resetting for subsequent transport cycles. Efforts in this area will be advanced by establishing the means to image transport dynamics in single LeuT molecules reconstituted into proteoliposomes, so that distinct ion and substrate concentrations can be maintained on either side of the bilayer. Experiments of this kind will greatly aid in the exploration of steps related to the return of the unloaded transporter, such as the role of protons^42,43^ and internal K^+^ ions^44^, the functional importance of each Na^+^ binding site^31,44^, and even the impact of the membrane itself on LeuT conformation^32,45^.

## Methods

### Protein expression and purification

LeuT variants were expressed in *E. coli* C41 (DE3) using pQO18-TEV vector derivatives with the mutations H7C/R86C (intracellular) or K239C/H480C (extracellular) as previously described^15^. Protein solubilized in n-dodecyl β-D-maltopyranoside (DDM) was purified using a Ni^2+^ Sepharose 6 FastFlow column (GE Healthcare). LeuT was labeled with maleimide-activated Cy3 and Cy5 (parent dyes, GE Healthcare) or LD550 and LD650 (Lumidyne Technologies) at a 1:1.5 molar ratio (200 µM total) for 1 h at 4 °C, followed by size exclusion chromatography using a Superdex 200 16/60 column. Labeled LeuT retained activity compared to wild-type protein (Supplementary Fig. 1).

### smFRET imaging experiments

Fluorescence experiments were performed using a prism-based total internal reflection fluorescence (TIRF) microscope as previously described^15,16^. Microfluidic imaging chambers passivated with a mixture of PEG and biotin-PEG were prepared with 0.8 µM streptavidin (Invitrogen) and 4 nM biotin-*tris*-(NTA-Ni^2+^)^46^. Fluorophore labeled, His-tagged LeuT molecules were reversibly immobilized to the surface-associated Ni^2+^ atoms. LD550 (Cy3) fluorophores were excited by the evanescent wave generated by total internal reflection (TIR) of a 532 nm diode-pumped solid state laser (Opus, Laser Quantum). Photons emitted from LD550 (Cy3) and LD650 (Cy5) were collected using a 1.27 NA 60 × PlanApo water-immersion objective (Nikon) and a MultiCam-LS device (Cairn) with a T635lpxr-UF2 dichroic mirror (Chroma) to separate the spectral channels onto two synchronized sCMOS cameras (Flash 4.0 v2, Hamamatsu). Fluorescence data were acquired using custom software implemented in LabView (National Instruments) at 10 frames per second (100 ms time resolution).

Unless otherwise specified, all experiments were performed at 25 °C in buffer containing 50 mM Tris/Mes (pH 7.0), 10% glycerol, 1 mM DDM (Anatrace), 1 mM 2-mercaptoethanol and 200 mM total salt (KCl and NaCl, as specified). An oxygen-scavenging environment consisting of 0.2 units per ml purified glucose oxidase (Sigma G2133), 1.8 units per μl purified catalase (Sigma C40), 0.1% (v/v) glucose was used in all experiments to minimize photobleaching. Catalase was purified by gel filtration before use due to evidence of contaminating concentrations of one or more small molecules in the commercial source that shifted the apparent Na^+^-binding affinity in the absence of substrates (compare Supplementary Fig. 1g and Fig. 2f).

Analysis of single-molecule fluorescence data was performed using custom software written in MATLAB (MathWorks)^22^. Spectral bleed-through from the donor to the acceptor channel was corrected by subtracting a set fraction (0.165) of the donor intensity from the acceptor. Due to the use of a different dichroic mirror (see above), corrections for acceptor to donor crosstalk^15^ were not necessary. The apparent brightness of the donor and acceptor fluorophores^21^ was approximately equal (*γ* ≈ 1.0) and did not change between experiments, making corrections unnecessary^15^. FRET traces were calculated as $E_{\rm FRET} = I_{\rm A}/(I_{\rm A} + I_{\rm D})$, where *I*_A_ and *I*_D_ are the donor and acceptor fluorescence traces, and set to zero whenever the donor was in the dark state. A subset of the acquired traces was selected for further analysis using the following criteria: (1) single-step donor photobleaching, (2) SNR_Background_ ≥ 15, (3) SNR_Signal_ ≥ 4, (4) < 4 donor blinking events, and (5) FRET efficiency above 0.15 for at least 300 frames (30 s). SNR_Background_ (30:1 on average) is defined as the signal magnitude relative to background noise. SNR_Signal_ (12:1 on average) is the signal magnitude relative to noise within the fluorescence trace prior to photobleaching, which includes all noise sources, including photon statistics and photophysical noise, in addition to background noise. Thresholds for SNR_Background_ and SNR_Signal_ were chosen to robustly and specifically remove a small fraction of aberrant and/or high noise traces. All conditions reported here contain a minimum of 800 selected molecules per repeat for a total of approximately 160,000 selected traces in all panels. Replicates (*n*) are defined as data acquired from independent immobilizations of LeuT, generally performed on separate days with newly prepared buffer solutions and frozen aliquots obtained from a single preparation of LeuT.

Initial model parameters were obtained by running ebFRET^29^ with approximately 1500 automatically selected traces truncated to remove dark states. Traces obtained in the presence of intermediate Na^+^ and Ala concentrations were utilized to ensure that all functionally relevant states are readily sampled. Iterations were stopped when the evidence lower bound converged within 1 × 10^−6^. smFRET trajectories were then idealized using the segmental K-means algorithm^47^ using the initial model parameters derived from ebFRET. Traces with FRET values more than one standard deviation from the model values (~30%), but otherwise similar behavior, were removed from analysis to improve idealization and histogram quality.

On both sides of LeuT, direct transitions between low-FRET and high-FRET states are observed without interleaving dwells in intermediate-FRET states. Such transitions may reflect instances in which intermediate-FRET dwells are too short to be captured or alternative routes between “open” and “closed” states.

### Scintillation proximity-based binding studies

We bound ^3^H-leucine (140 Ci mmol^–1^; Moravek) to purified LeuT-variants using the scintillation proximity assay (SPA) as described^17,18^ with 25 ng of purified protein per assay in buffer composed of 50 mM Tris/Mes (pH 8.0), 100 mM NaCl, 1 mM TCEP, 20% glycerol and 1 mM DDM.

### Figure preparation

All structure figures were made using PyMOL^48^.

### Structural models

Structural models of LeuT in the inward-facing intermediate state were constructed with multi-template homology modeling using MODELLER^49^. The templates used in this established protocol represent Macrostates 8, 11, 4, and 1 (respectively designated in Supplementary Fig. 9 as Model States 1, 2, 3, and 4) defined in the published kinetic model for Na^+^ release from hDAT^35^. For each macrostate we selected either 300 random structures corresponding to that macrostate (or all structures if fewer than 300 were available), and generated 100 LeuT homology models corresponding to each macrostate. The distribution of H7/R86 and R11/R86 C_β_ carbon distances was calculated for all models in each macrostate.

### Estimation of the maximum allosteric destabilization

In order to estimate the maximum potential destabilization of the doubly open state due to allosteric coupling between the gates, we utilized thermodynamic coupling function (TCF) theory^37^. In TCF theory one considers a pair of reaction coordinates or collective variables, *X* and *Y*, and then calculates the thermodynamic coupling, ΔΔ*A*(*x*,*y*), at each point (*x*,*y*) in the bivariate state space,

1 $${\mathrm{\Delta \Delta {\it A}}}\left( {x,y} \right) = - kT{\mathrm{log}}\left( {\frac{{{{p}}\left( {{{x,y}}} \right)}}{{{{p}}\left( {{x}} \right){{p}}\left( {{y}} \right)}}} \right),$$

where *p*(*x*), *p*(*y*), and *p*(*x*,*y*) are the marginal and joint probabilities of the states. We estimated the TCF between the low-FRET states on each site, ΔΔ*A*(low_IC_, low_EC_), as

2 $${\mathrm{\Delta \Delta {\it A}}}\left( {{\mathrm{low}}_{{\mathrm{IC}}},{\mathrm{low}}_{\rm EC}} \right) \approx -kT {\mathrm{log}}\left( {\frac{{{{p}}\prime\left( {{\mathrm{low}}_{{\mathrm{IC}}},{\mathrm{low}}_{{\mathrm{EC}}}} \right)}}{{{{p}}\left( {{\mathrm{low}}_{{\mathrm{IC}}}} \right){{p}}\left( {{\mathrm{low}}_{{\mathrm{EC}}}} \right)}}} \right),$$

where the marginal probabilities were estimated from the occupancies in the smFRET experiments, and the joint probability was taken to be

3 $${{p}}\prime\left( {{\mathrm{low}}_{{\mathrm{IC}}},{\mathrm{low}}_{{\mathrm{EC}}}} \right) = {\mathrm{min}}\left( {{{p}}\left( {{\mathrm{low}}_{{\mathrm{IC}}}} \right),{{p}}\left( {{\mathrm{low}}_{{\mathrm{EC}}}} \right)} \right) - (1 - {\mathrm{max}}\left( {{{p}}\left( {{\mathrm{low}}_{{\mathrm{IC}}}} \right),{{p}}\left( {{\mathrm{low}}_{{\mathrm{EC}}}} \right)} \right)).$$

Because the probability of both low-FRET states is greater than 0.5, Eq (3) ensures that the estimated joint probability of the low-FRET states is minimized given the marginal probabilities of the low-FRET states.

### Code availability

The full source code of SPARTAN^22^, which was used for all analysis of smFRET data, is publicly available: http://www.scottcblanchardlab.com/software.

### Data availability

The data that support the findings of this study are available from the corresponding authors upon reasonable request.

## Electronic supplementary material

Supplementary Information

## References

[1] Yamashita A, Singh SK, Kawate T, Jin Y, Gouaux E (2005). Crystal structure of a bacterial homologue of Na^+^/Cl^−^-dependent neurotransmitter transporters. Nature.

[2] Forrest LR (2008). Mechanism for alternating access in neurotransmitter transporters. Proc. Natl Acad. Sci. USA.

[3] Torres GE, Gainetdinov RR, Caron MG (2003). Plasma membrane monoamine transporters: structure, regulation and function. Nat. Rev. Neurosci..

[4] Iversen L (2006). Neurotransmitter transporters and their impact on the development of psychopharmacology. Br. J. Pharmacol..

[5] Amara SG, Sonders MS (1998). Neurotransmitter transporters as molecular targets for addictive drugs. Drug Alcohol Depend..

[6] Freyberg Z (2016). Mechanisms of amphetamine action illuminated through optical monitoring of dopamine synaptic vesicles in Drosophila brain. Nat. Commun..

[7] Krishnamurthy H, Gouaux E (2012). X-ray structures of LeuT in substrate-free outward-open and apo inward-open states. Nature.

[8] Coleman JA, Green EM, Gouaux E (2016). X-ray structures and mechanism of the human serotonin transporter. Nature.

[9] Penmatsa A, Wang KH, Gouaux E (2013). X-ray structure of dopamine transporter elucidates antidepressant mechanism. Nature.

[10] Loland CJ (2015). The use of LeuT as a model in elucidating binding sites for substrates and inhibitors in neurotransmitter transporters. Biochim. Biophys. Acta.

[11] Krishnamurthy H, Piscitelli CL, Gouaux E (2009). Unlocking the molecular secrets of sodium-coupled transporters. Nature.

[12] Jardetzky O (1966). Simple allosteric model for membrane pumps. Nature.

[13] Shi YG (2013). Common folds and transport mechanisms of secondary active transporters. Annu Rev. Biophys..

[14] Smirnova I, Kasho V, Kaback HR (2011). Lactose permease and the alternating access mechanism. Biochemistry.

[15] Zhao Y (2010). Single-molecule dynamics of gating in a neurotransmitter transporter homologue. Nature.

[16] Zhao Y (2011). Substrate-modulated gating dynamics in a Na^+^-coupled neurotransmitter transporter homologue. Nature.

[17] Shi L, Quick M, Zhao Y, Weinstein H, Javitch JA (2008). The mechanism of a neurotransmitter:sodium symporter–inward release of Na^+^ and substrate is triggered by substrate in a second binding site. Mol. Cell.

[18] Quick M, Shi L, Zehnpfennig B, Weinstein H, Javitch JA (2012). Experimental conditions can obscure the second high-affinity site in LeuT. Nat. Struct. Mol. Biol..

[19] Nasr ML, Singh SK (2014). Radioligand binding to nanodisc-reconstituted membrane transporters assessed by the scintillation proximity assay. Biochemistry.

[20] Adhikary S (2017). Conformational dynamics of a neurotransmitter:sodium symporter in a lipid bilayer. Proc. Natl Acad. Sci. USA.

[21] Roy R, Hohng S, Ha T (2008). A practical guide to single-molecule FRET. Nat. Methods.

[22] Juette MF (2016). Single-molecule imaging of non-equilibrium molecular ensembles on the millisecond timescale. Nat. Methods.

[23] LeVine MV, Cuendet MA, Khelashvili G, Weinstein H (2016). Allosteric mechanisms of molecular machines at the membrane: transport by sodium-coupled symporters. Chem. Rev..

[24] Kazmier K, Claxton DP, McHaourab HS (2016). Alternating access mechanisms of LeuT-fold transporters: trailblazing towards the promised energy landscapes. Curr. Opin. Struct. Biol..

[25] Kazmier K (2014). Conformational dynamics of ligand-dependent alternating access in LeuT. Nat. Struct. Mol. Biol..

[26] Zheng QS (2014). Ultra-stable organic fluorophores for single-molecule research. Chem. Soc. Rev..

[27] Juette MF (2014). The bright future of single-molecule fluorescence imaging. Curr. Opin. Chem. Biol..

[28] Akyuz N (2015). Transport domain unlocking sets the uptake rate of an aspartate transporter. Nature.

[29] van de Meent JW, Bronson JE, Wiggins CH, Gonzalez RL (2014). Empirical Bayes methods enable advanced population-level analyses of single-molecule FRET experiments. Biophys. J..

[30] Singh SK, Piscitelli CL, Yamashita A, Gouaux E (2008). A competitive inhibitor traps LeuT in an open-to-out conformation. Science.

[31] Tavoulari S (2016). Two Na^+^ sites control conformational change in a neurotransmitter transporter homolog. J. Biol. Chem..

[32] Sohail A (2016). The environment shapes the inner vestibule of LeuT. PLoS Comput. Biol..

[33] Stolzenberg S, Michino M, LeVine MV, Weinstein H, Shi L (2016). Computational approaches to detect allosteric pathways in transmembrane molecular machines. Biochim. Biophys. Acta.

[34] Malinauskaite L (2014). A mechanism for intracellular release of Na^+^ by neurotransmitter/sodium symporters. Nat. Struct. Mol. Biol..

[35] Razavi AM, Khelashvili G, Weinstein H (2017). A Markov state-based quantitative kinetic model of sodium release from the dopamine transporter. Sci. Rep..

[36] Stolzenberg S (2015). Mechanism of the association between Na^+^ binding and conformations at the intracellular gate in neurotransmitter:sodium symporters. J. Biol. Chem..

[37] Cuendet MA, Weinstein H, LeVine MV (2016). The allostery landscape: quantifying thermodynamic couplings in biomolecular systems. J. Chem. Theory Comput..

[38] Billesbolle CB (2015). Substrate-induced unlocking of the inner gate determines the catalytic efficiency of a neurotransmitter:sodium symporter. J. Biol. Chem..

[39] Shaikh SA (2013). Visualizing functional motions of membrane transporters with molecular dynamics simulations. Biochemistry.

[40] Cheng MH, Bahar I (2014). Complete mapping of substrate translocation highlights the role of LeuT N-terminal segment in regulating transport cycle. PLoS Comput. Biol..

[41] Gur M, Zomot E, Cheng MH, Bahar I (2015). Energy landscape of LeuT from molecular simulations. J. Chem. Phys..

[42] Zhao Y (2010). Substrate-dependent proton antiport in neurotransmitter:sodium symporters. Nat. Chem. Biol..

[43] Khelashvili G (2016). Conformational dynamics on the extracellular side of LeuT controlled by Na^+^ and K^+^ Ions and the protonation state of Glu290. J. Biol. Chem..

[44] Billesbolle CB (2016). Transition metal ion FRET uncovers K^+^ regulation of a neurotransmitter/sodium symporter. Nat. Commun..

[45] Mondal S, Khelashvili G, Shi L, Weinstein H (2013). The cost of living in the membrane: a case study of hydrophobic mismatch for the multi-segment protein LeuT. Chem. Phys. Lipids.

[46] Lata S, Reichel A, Brock R, Tampe R, Piehler J (2005). High-affinity adaptors for switchable recognition of histidine-tagged proteins. J. Am. Chem. Soc..

[47] Qin F (2004). Restoration of single-channel currents using the segmental k-means method based on hidden Markov modeling. Biophys. J..

[48] Schrodinger, L. L. C. *The PyMOL Molecular Graphics System, Version**1.8 *(Schrödinger, LLC, New York, NY, 2015).

[49] Webb B, Sali A (2014). Comparative protein structure modeling using MODELLER. Curr. Protoc. Bioinforma..

[50] Haran G (2004). Noise reduction in single-molecule fluorescence trajectories of folding proteins. Chem. Phys..

