# Supplementary material for: A partially-open inward-facing intermediate conformation of LeuT is associated with Na+ release and substrate transport
Source: Nat Commun. 2018 Jan 15;9:230. doi: 10.1038/s41467-017-02202-y (PMC5768729; doi:10.1038/s41467-017-02202-y)
Supplement: Supplementary file 1 — Supplementary Information [file 41467_2017_2202_MOESM1_ESM.pdf]

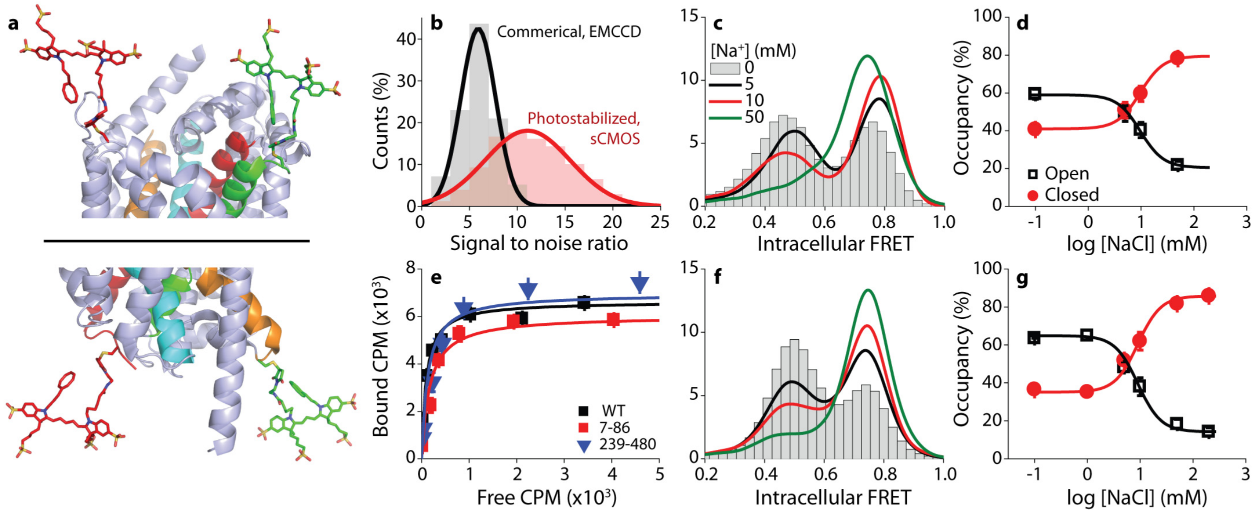

Supplementary Figure 1. Comparison of commercially available and photostabilized fluorophores.

(**a**) Cartoon representation of LeuT (PDB code 2A65) showing the self-healing LD550 (green) and LD650 (red) fluorophores attached at labeling positions K239C and H480C on the extracellular side (top) and H7C and R86C on the intracellular side (bottom). (**b**) Comparison of the ratios of signal magnitude to noise within the total fluorescence intensity prior to photobleaching (SNR-$_{\text{Signal}}$) from experiments using EMCCD cameras to image LeuT labeled with commercially available fluorophores at 160 ms time resolution (gray bars), as previously described[1], or self-healing fluorophores using sCMOS cameras, and 100 ms time resolution (red bars), in the absence of Na^+ and substrates. (**c**) Effect of various concentrations of Na^+ on FRET distributions from 7C/86C-LeuT labeled with parent fluorophores. (**d**) Fraction occupancy in each state from panel c. Lines are fits to dose-response functions with Hill coefficients fixed at 2.0. (**e**) Concentration dependence and binding stoichiometry, as determined by scintillation proximity assay[2] using ^{3}H-Leu binding to wild-type (■), H7C/R86C (■), or K239C/H480C (▼) LeuT labeled with self-healing fluorophores. (**f-g**) As in panels c and d, but with self-healing fluorophores. Error bars are mean ± s.e.m. of *n*=3 independent experiments.

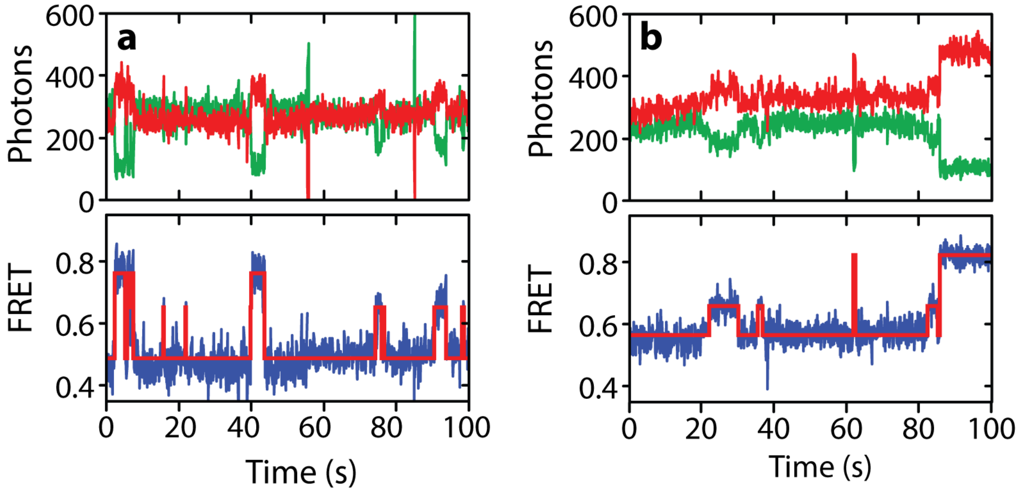

Supplementary Figure 2. FRET state assignments.
LeuT labeled at (**a**) extracellular sites (K239C/H480C) and (**b**) intracellular sites (H7C/R86C) was imaged in the absence of Na^+ and substrate. Representative single molecule fluorescence (donor in green, acceptor in red) and FRET (blue) traces highlight the three distinct FRET states (red dotted lines) observed from both structural perspectives.

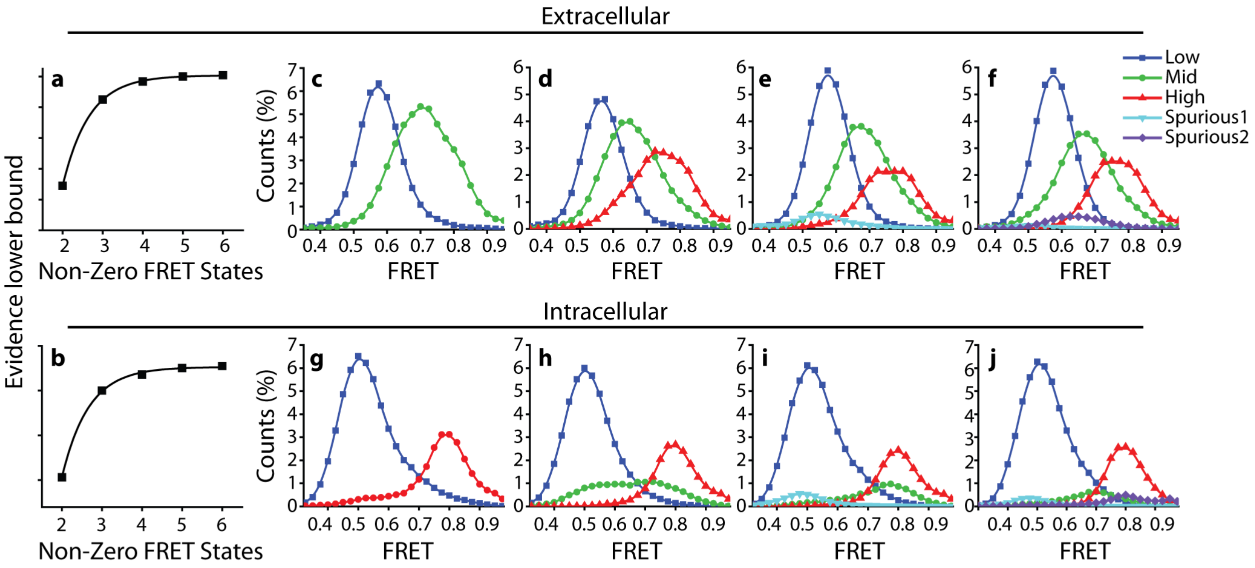

Supplementary Figure 3. Determining model parameters with ebFRET.
ebFRET[3] was used to analyze approximately 1,500 automatically selected traces, acquired in the presence of 5 mM Na^+ and 60 μM Ala, to determine the number of non-zero FRET states in an unbiased manner and provide initial estimates of model parameters. The analysis was repeated specifying two to six model states. (**a**,**b**) Evidence lower bound, indicating the level of support for the indicated number of model states, is shown for data acquired from the (**a**) extracellular and (**b**) intracellular structural perspectives. Histograms of FRET values in each assigned state are shown for analysis with (**c**) two, (**d**) three, (**e**) four, and (**f**) five non-zero FRET states on the extracellular side and (**g-j**) on the intracellular side.

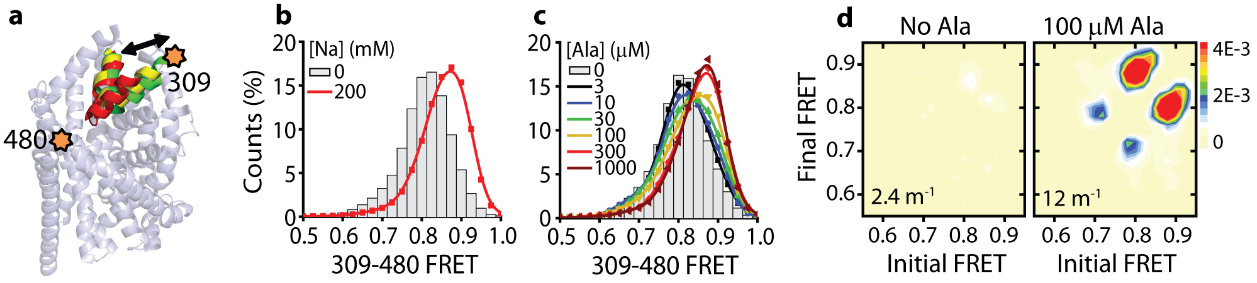

Supplementary Figure 4. Effects of Na$^+$ and Ala on FRET between EL4 and TM12.
(**a**) Schematic of A309C/H480C-LeuT labeled with LD550 and LD650 fluorophores (stars). The positions of EL4 are highlighted from crystal structures of LeuT in various conformations to show the likely direction of motion observed by smFRET, with relatively closed (red, 3TT3), intermediate (yellow, 2A65), and open (3TT1, green) relative positions of EL4. (**b**) FRET histogram in the absence of ligands (gray bars) and in the presence of 200 mM Na$^+$ (red line). (**c**) FRET histograms in the presence of 5 mM Na$^+$ and the indicated concentration of Ala. (**d**) Transition density plots in the absence of substrate (left) and in the presence of 100 μM Ala (both in the presence of 5 mM Na$^+$). The numbers in the lower-left corners are the average number of transitions per minute of imaging time. Scale bar at right (transitions per second per bin). The analysis includes a total of 7,915 selected traces.

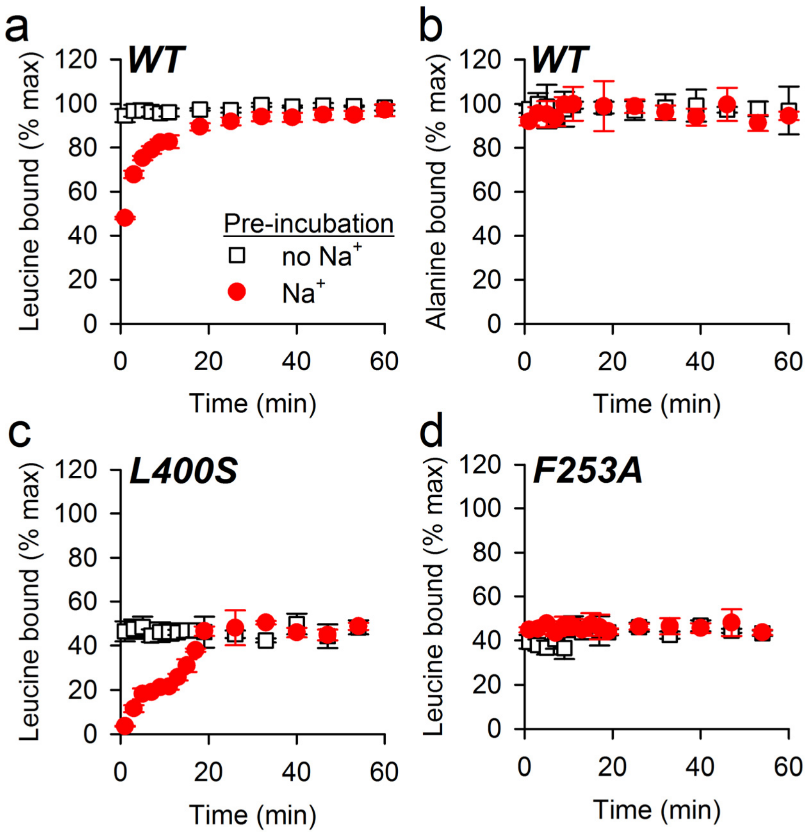

Supplementary Figure 5. Pre-incubation of LeuT with Na^+ modulates substrate binding. The time course of binding of (**a,c,d**) ^{3}H-Leu (100 nM) or (**b**) ^{3}H-Ala (500 nM) to LeuT wild type (WT; **a,b**) or LeuT-L400S (**c**) or -F253A (**d**) in the absence (open black squares) or presence (filled red circles) of Na^+ was measured using the scintillation proximity assay[4] (see **Methods**). Data are shown as mean ± s.e.m. of three technical replicates of a representative experiment performed in parallel (experiments with individual mutants were repeated ≥ 2) and normalized with regard to the steady state level of LeuT-WT.

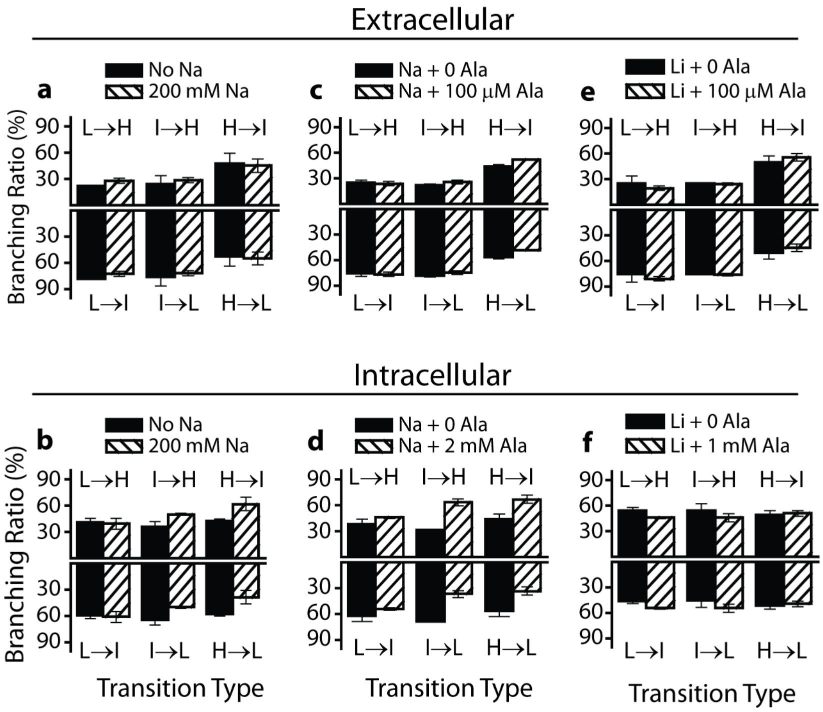

Supplementary Figure 6. Transition branching ratios. Shown in each panel is the fraction of dwells in the low (L, left), intermediate (I, middle), and high (H, right) FRET states that transition to the indicated final states (e.g., L→H is the fraction of dwells in the low-FRET state that transition to the high-FRET instead of intermediate-FRET state) from experiments imaging the (**a,c,e**) extracellular or (**b,d,f**) intracellular face of LeuT. (**a,b**) Experiments imaging LeuT in the absence (solid bars) and presence (shaded bars) of 200 mM Na^+, corresponding to Figure 2. (**c,d**) Experiments imaging LeuT in the presence of 5 mM Na^+ and the absence (solid bars) and presence (shaded bars) of the indicated concentration of Ala, corresponding to Figure 3. (**e,f**) Experiments imaging LeuT in the presence of 100 mM Li^+ and the absence (solid bars) and presence (shaded bars) of the indicated concentration of Ala, corresponding to Figure 4. Error bars are mean ± s.d. of two independent experiments.

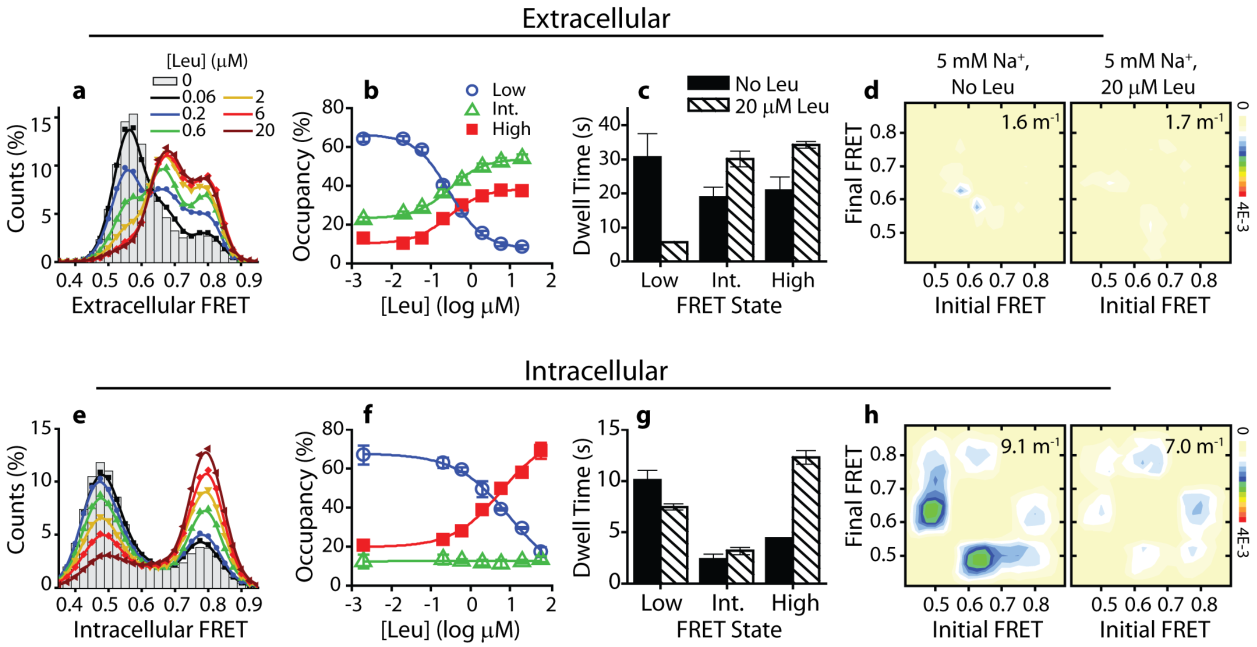

Supplementary Figure 7. Leu favors closed states and slows dynamics.
(**a**) FRET histograms of intracellular labeled LeuT in the presence of 5 mM Na^+ and the indicated concentration of Leu. (**b**) Fractional occupancy in low (○), intermediate (△), and high (■) extracellular FRET states as a function of Leu concentrations. (**c**) Mean dwell times in each extracellular FRET state in the absence (solid bars) and presence (shaded bars) of 20 μM Leu. (**d**) Transition density plots in the absence (left) and in the presence (right) of 20 μM Leu, with average transitions rate in the upper-right corner of each. (**e-h**) As in panels a-d, but from the intracellular labeling perspective. Lines in panels b and f are fits to Hill equations with slopes fixed at 1.0 and EC_{50} of 0.28 and 8.0 μM, respectively. Error bars in all panels are the mean ± s.d. of two independent experiments, including a total of 14,113 and 8,949 molecules for extracellular and intracellular perspectives, respectively.

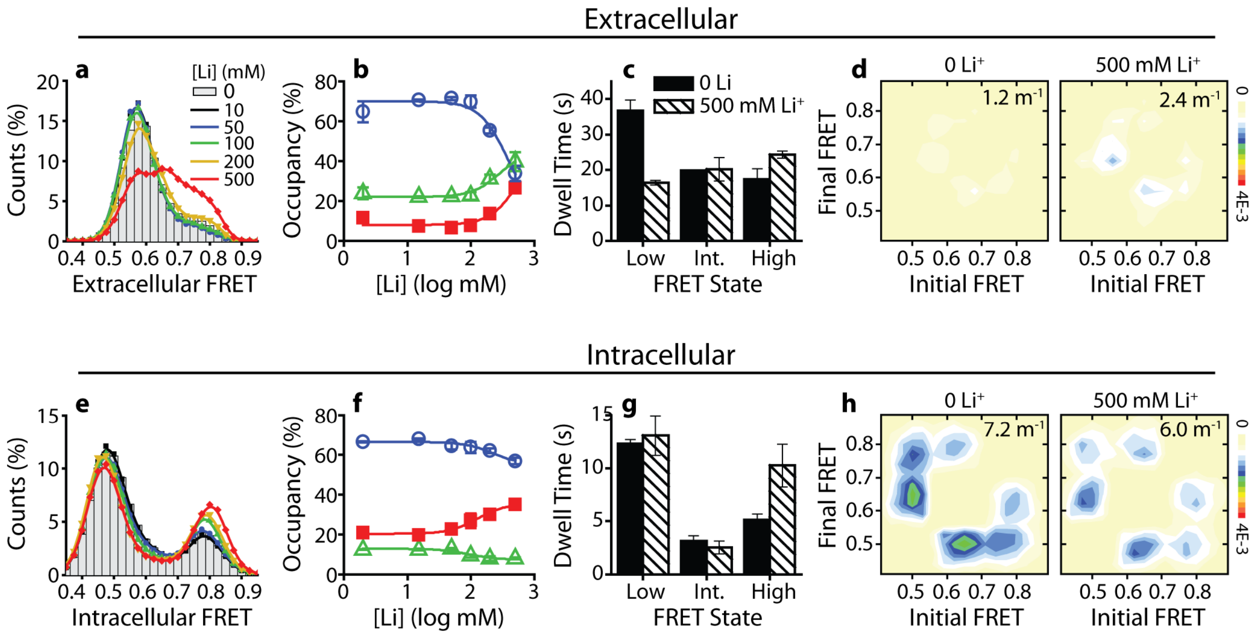

Supplementary Figure 8. Li$^+$ decreases low-FRET state occupancy at both faces.
(**a**) FRET histograms of extracellular labeled LeuT in the absence of ligands (gray bars), and in the presence of the indicated concentration of Li$^+$ (lines). (**b**) Overall occupancy in low (○) intermediate (△), and high (■) extracellular FRET states as a function of Li$^+$ concentration. (**c**) Mean dwell times in the absence (solid bars) and presence (shaded bars) of 500 mM Li$^+$. (**d**) Transition density plots in the absence (left) and presence (right) of 500 mM Li$^+$. (**e-h**) As in panels a-d, but from intracellular labeling perspective. Lines are fits to Hill equation with slopes fixed at 2.0 and EC_{50} of 400 mM outside and 140 mM inside. Due to incomplete saturation, the calculated EC_{50} values represent lower-bound estimates. Error are mean ± s.d. of two independent experiments, including a total of 10,822 and 10,666 molecules for extracellular and intracellular perspectives, respectively.

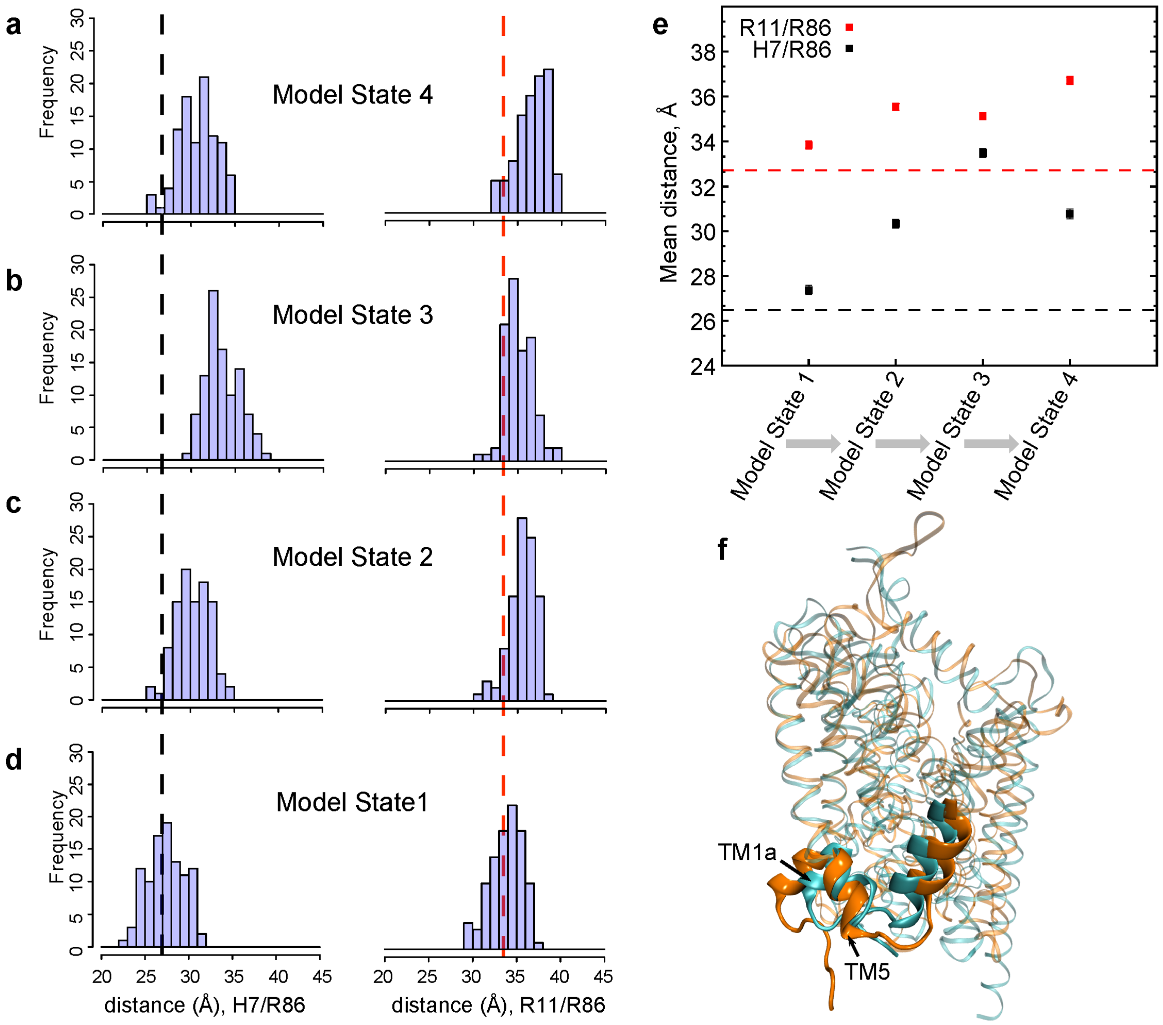

Supplementary Figure 9: Markov state modeling of LeuT intermediate states. (**a-d**) Histograms of the Cβ-Cβ H7/R86 (*left column*) and R11/R86 (*right column*) distances in the ensemble of LeuT structures obtained from homology modeling based on the previously published kinetic model for Na^+/Na2 release in hDAT (see Methods). Model States 1, 2, 3, and 4 correspond, respectively, to hDAT macrostates 8, 11, 4 and 1 as previously described[5]. (**e**) The progression of mean distances (from the histograms in panels a-d) in the transition from the Na2 occupied, inward closed state (Macrostate 1) to the Na2 released intermediate inward open (Macrostate 4). Black and red dotted lines in panels a-e represent the 7-86 and 11-86 distances, respectively, in the crystal structure of LeuT in the occluded state (PDB ID: 2A65). (**f**) Superposition of the 2A65 structure (*cyan*) on a model LeuT structure corresponding to the most probable configuration of the "Model State 4" ensemble (*orange*) highlighting predicted structural changes in LeuT upon Na^+/Na2 release: outward displacement of TM1a (residues 5-17) and unwinding of intracellular region of TM5 (residues 175-195).

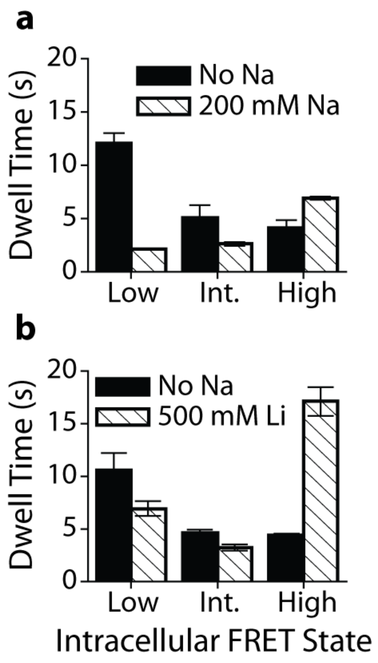

Supplementary Figure 10. Effect of Na^+ and Li^+ in the presence of Ala on intracellular dynamics. (**a**) Mean dwell times in the intracellular low, intermediate, and high-FRET states from experiments imaging LeuT in the presence of 250 µM Ala and the absence (solid bars) and presence (shaded bars) of 200 mM Na^+. (**b**) As in panel a, but in the absence (solid bars) and presence (shaded bars) of 500 mM Li^+. Experiments correspond to those in Figure 4. Error bars are mean ± s.d. of two independent experiments.

Sites	FRET Values			Inter-dye Distance (Å)			Crystal Distance (Å)		
	**Low**	**Mid**	**High**	**Low/ Mid**	**Mid/ High**	**Low/ High**	**Open/ Compact**	**Compact/ Closed**	**Open/ Closed**
**239/480**	0.55	0.67	0.79	4.6	5.1	9.6	3.7	5.5	9.2
**7/86**	0.47	0.63	0.79	6.1	6.6	12	N/A	N/A	24*
**309/480**	0.72	0.80	0.88	3.5	4.5	8.0	5.5	6.6	12.1

Supplementary Table 1. Comparison of distance changes predicted from FRET values versus crystal structures at both faces of LeuT. N/A, no structure available for comparison. *Measured between residues 11 and 86 because residue 7 is not resolved in the crystal structure. Inter-dye distances were calculated from FRET values according to the equation: $R = R_0 \sqrt[6]{(1-E)/E}$, where R_0 = 58.4 Å and E is the FRET efficiency.

Supplementary References:

1 Zhao, Y. *et al.* Substrate-modulated gating dynamics in a Na^+-coupled neurotransmitter transporter homologue. *Nature* **474**, 109-113, (2011).

2 Quick, M. & Javitch, J. A. Monitoring the function of membrane transport proteins in detergent-solubilized form. *Proc Natl Acad Sci U S A* **104**, 3603-3608, (2007).

3 van de Meent, J. W., Bronson, J. E., Wiggins, C. H. & Gonzalez, R. L., Jr. Empirical Bayes methods enable advanced population-level analyses of single-molecule FRET experiments. *Biophys J* **106**, 1327-1337, (2014).

4 Shi, L., Quick, M., Zhao, Y., Weinstein, H. & Javitch, J. A. The mechanism of a neurotransmitter:sodium symporter--inward release of Na^+ and substrate is triggered by substrate in a second binding site. *Mol Cell* **30**, 667-677, (2008).

5 Razavi, A. M., Khelashvili, G. & Weinstein, H. A Markov State-based Quantitative Kinetic Model of Sodium Release from the Dopamine Transporter. *Sci Rep* **7**, 40076, (2017).
